# Taxon- and Growth Phase-Specific Antioxidant Production by Chlorophyte, Bacillariophyte, and Haptophyte Strains Isolated From Tropical Waters

**DOI:** 10.3389/fbioe.2020.581628

**Published:** 2020-11-23

**Authors:** Norazira Abdu Rahman, Tomoyo Katayama, Mohd Effendy Abd Wahid, Nor Azman Kasan, Helena Khatoon, Yuichiro Yamada, Kazutaka Takahashi

**Affiliations:** ^1^Department of Aquatic Bioscience, Graduate School of Agricultural and Life Sciences, The University of Tokyo, Tokyo, Japan; ^2^Institute of Marine Biotechnology, Universiti Malaysia Terengganu, Kuala Terengganu, Malaysia; ^3^School of Fisheries and Aquaculture Sciences, Universiti Malaysia Terengganu, Kuala Terengganu, Malaysia; ^4^Institute of Tropical Aquaculture and Fisheries, Universiti Malaysia Terengganu, Kuala Terengganu, Malaysia; ^5^Department of Aquaculture, Faculty of Fisheries, Chittagong Veterinary and Animal Sciences University, Chittagong, Bangladesh; ^6^School of Marine Biosciences, Kitasato University, Kanagawa, Japan

**Keywords:** phenolic, carotenoid, fatty acids, growth phase, antioxidant capacity, micro algae

## Abstract

Antioxidants found in microalgae play an essential role in both animals and humans, against various diseases and aging processes by protecting cells from oxidative damage. In this study, 26 indigenous tropical marine microalgae were screened. Out of the 26 screened strains, 10 were selected and were further investigated for their natural antioxidant compounds which include carotenoids, phenolics, and fatty acids collected in their exponential and stationary phases. The antioxidant capacity was also evaluated by a total of four assays, which include ABTS, DPPH, superoxide radical (O_2_^•–^) scavenging capacity, and nitric oxide (•NO^–^) scavenging capacity. This study revealed that the antioxidant capacity of the microalgae varied between divisions, strains, and growth phase and was also related to the content of antioxidant compounds present in the cells. Carotenoids and phenolics were found to be the major contributors to the antioxidant capacity, followed by polyunsaturated fatty acids linoleic acid (LA), eicosapentaenoic acid (EPA), arachidonic acid (ARA), and docosahexaenoic acid (DHA) compared to other fatty acids. The antioxidant capacity of the selected bacillariophytes and haptophytes was found to be positively correlated to phenolic (*R*^2^-value = 0.623, 0.714, and 0.786 with ABTS, DPPH, and •NO^–^) under exponential phase, and to carotenoid fucoxanthin and β-carotene (R^2^ value = 0.530, 0.581 with ABTS, and 0.710, 0.795 with O_2_^•–^) under stationary phase. Meanwhile, antioxidant capacity of chlorophyte strains was positively correlated with lutein, β-carotene and zeaxanthin under the exponential phase (R^2^ value = 0.615, 0.615, 0.507 with ABTS, and R^2^ value = 0.794, 0.659, and 0.509 with •NO^–^). In the stationary phase, chlorophyte strains were positively correlated with violaxanthin (0.755 with •NO^–^), neoxanthin (0.623 with DPPH, 0.610 with •NO^–^), and lutein (0.582 with •NO^–^). This study showed that antioxidant capacity and related antioxidant compound production of tropical microalgae strains are growth phase-dependent. The results can be used to improve the microalgal antioxidant compound production for application in pharmaceutical, nutraceutical, food, and feed industry.

## Introduction

Highly reactive free radicals, including reactive oxygen species (ROS) and reactive nitrogen species (RNS), such as hydroxyl radicals (•OH), superoxide anions (O_2_^•–^), and hydrogen peroxides (H_2_O_2_), are generated as a part of normal cellular metabolism in both humans and animals. Under normal conditions, these radicals are part of the cellular redox signaling and immune function and are readily converted into a safer intermediate (Pham-Huy et al., 2008). However, under abiotic or biotic stress, the imbalance between the rate of radical production and antioxidant defense may induce oxidative stress, leading to the oxidation of proteins, lipids, DNA, and eventually, cell death (Halliwell, 2007). An organism can counteract this situation by various enzymatic and non-enzymatic mechanisms, including antioxidant compounds such as carotenoids and polyphenols (Oroian and Escriche, 2015). An antioxidant is defined as any substance that delays or inhibits the oxidation of substrates even when present at low concentrations compared to the oxidizable substrate (Gutteridge, 1995). Butylated hydroxyanisole (BHA) and butylated hydroxytoluene (BHT) are two widely used synthetic antioxidants in the food and feed industry. However, increasing concerns about their potential carcinogenic and toxic effects have led to a broader search for a natural and sustainable source of antioxidants (Shebis et al., 2013).

Microalgae are natural sources of antioxidants, which have an excellent ability to accumulate various intracellular valuable bioactive compounds (Venkatesan et al., 2015; Sansone and Brunet, 2019). In particular, their fast growth rate and high productivity under normal or stressful conditions make them an attractive candidate for the sustainable alternative of antioxidant sources with high nutritional value (Bulut et al., 2019). It is well-known that the concentrations of microalgal bioactive compounds vary widely depending on the original habitat, species, strains, and growth phases, which may influence the differences in the antioxidant properties of certain microalgae. Microalgal flora in tropical waters has a high potential to accommodate natural antioxidants because their habitat is characterized by high temperature, light, and ultraviolet radiation almost all year round, creating harsh environmental conditions that require defense mechanisms to survive against oxidative stress. Therefore, screening for indigenous tropical microalgae, which have adapted to the harsh environment, would be beneficial in the search for new strains having high antioxidant properties.

Various bioactive compounds found in microalgae play an essential role in protection against oxidative stress and damage. Some of the most important and well-known antioxidants from microalgae include carotenoids and phenolics (Safafar et al., 2015). Both compounds are considered as potent non-enzymatic antioxidants capable of protecting against oxidative damage by converting the radical into a safer by-product. The complex ringed chain molecules of carotenoids enable them to absorb the energy of singlet oxygen radicals and delay the propagation of lipid peroxidation chain reactions, which can disintegrate the lipid membrane (Takaichi, 2011). Meanwhile, a phenolic compound may act as an antioxidant by single electron transfer (SAT) or hydrogen atom transfer (HAT) (Goiris et al., 2012). Recently, fatty acids, especially one with a high degree of unsaturation, have been shown to contribute to the antioxidant activity of various microalgae (Banskota et al., 2018). Nevertheless, very few studies have evaluated the correlation between the antioxidant activity and lipid or fatty acid composition of microalgal strains. According to Lavens and Sorgeloos (1996), microalgae growth phase is characterized into five phases which include lag (initial cell metabolism adaptation to growth), exponential (increased growth), declining growth rate (cell division slows down due to physical or chemical limitation), stationary (constant cell density) and death phase (due to depleted nutrient). Optimal harvesting of microalgae biomass for various industries is usually conducted at an exponential or stationary phase as these are the two phases with high cell biomass and overall compound productivity. However, so far, the antioxidant capacity of microalgae is usually determined using samples taken at either exponential or stationary phase. Thus, the results mostly represent a particular growth phase, without considering the differences in the antioxidant compound constitution among phases.

In this study, the results of the screening of 26 indigenous marine microalgae isolated from Malaysian water bodies for antioxidant production were presented. Being a country with high biodiversity and various unique water bodies, Malaysia is a promising habitat for millions of phytoplankton (Mazlan et al., 2005). However, only a few strains have been isolated and screened for antioxidant production (Natrah et al., 2007). As microalgal antioxidant production could vary depending on the locality, species, strains, and culture conditions, the antioxidant capacity of the microalgae was screened and compared between different taxonomic groups (Bacillariophyta, Chlorophyta, and Haptophyta) at different growth phases in relation to their bioactive compounds.

## Materials and Methods

### Molecular Identification

In this study, twenty-three strains newly isolated from several Malaysian water bodies were identified by molecular identification. The strains were originated from (1) Remis Beach, Selangor (N03°12′ E101°18′), (2) Teluk Ketapang Beach, Terengganu (N05°23′ E103°06′), and (3) Shrimp pond, Terengganu (N05°38′ E102°45′). For molecular identification, DNA was extracted from the microalgae cells using the DNeasy Plant Mini Kit (Qiagen, Germany). Prior to extraction, microalgal cell disruption was carried out using zirconium beads (EZ-Beads, AMR, Japan) to ensure the release of intracellular contents (especially DNA) into the medium. One milliliter of microalgal cells was bead-beaten for 2 min, centrifuged at 14,000 rpm for 1 min, and the supernatant was used for PCR amplification. For amplification of the SSU region of chlorophytes, the primer combinations used were NS1F-1650R, NS1F-1150R, 1170F-1650R, or PRIMER A-PRIMER B as described previously by Medlin et al. (1988) and Štenclová et al. (2017). For the amplification of the ITS region for the chlorophytes, the primers ITS1 and Pico-ITS4 adapted from Hadi et al. (2016) were used. For the bacillariophyte strain, the primer combination used for amplification of the SSU rDNA region was 1-F-1528-R (Medlin et al., 1988). All primer information are listed in Table 1. The amplicon, as determined by gel electrophoresis, was purified using the QIAquick PCR Purification Kit (Qiagen Genomics, United States) and then sequenced by Eurofins Genomics Inc. (Tokyo, Japan). For the microalgae species identification, the resulting SSU ITS rDNA sequences were aligned and analyzed using the BLAST algorithm at the National Centre for Biotechnology Information. Despite of its high antioxidant capacity, one of the screened bacillariophyte strain TRG8-01 was not included in further analysis as it was not successful to be identified even at genus level. The detail of the unidentified strain is shown in the Supplementary Materials.

**TABLE 1:** List of primers used for PCR amplification in the present study.

Gene	Name	Sequence	F/R	References
SSU	NS1F	GTAGTCATATGCTTGTCTC	F	Štenclová et al., 2017
	1150R	ACGCCTGGTGGTGCCCTTCCGT	R	Štenclová et al., 2017
	1170F	CTGTGGCTTAATTTGACTCAACACG	F	Štenclová et al., 2017
	1650R	TCACCAGCACACCCAAT	R	Štenclová et al., 2017
	PRIMER A	AACCTGGTTGATCCTGCCAGT	F	Aburai et al., 2013
	PRIMER B	TGATCCTTCTGCAGGTTCACCTAC	R	Aburai et al., 2013
	1-F	AACCTGGTTGATCCTGCCAGTA	F	Rimet et al., 2011
	1528-R	CTTCTGCAGGTTCACCTAC	R	Rimet et al., 2011
ITS	Fw_ITS1Pico	GGAAGGAGAAGTCGTAACAA	F	Hadi et al., 2016
	Rv_ITS4	TCCTCCGCTTATTGATATGC	R	Hadi et al., 2016

### Microalgae Strains and Screening Experiments

A total of 26 indigenous marine microalgae strains, including 22 of newly isolated strains and 4 established strains belonging to six algal classes, namely, Trebouxiophyceae, Chlorophyceae, Prasinophyceae, Bacillariophyceae, Mediophyceae, and Coccolithophyceae, were screened for their antioxidant capacity. Established strains of *Chaetoceros gracilis*, *Isochrysis galbana*, and *Chlorella vulgaris* were obtained from Universiti Putra Malaysia (Natrah et al., 2007; Goh et al., 2010). *Tetraselmis suecica* were obtained from the University Malaysia Terengganu. *Phaeodactylum tricornutum* (CCMP1327) was obtained from the Provosoli-Guillard National Center for Culture of Marine Phytoplankton, United States, and used as the control strain. This strain is known to be rich in fucoxanthin, a carotenoid known for its antioxidant activity (Goiris et al., 2012; Guo et al., 2016).

A pre-inoculum was cultivated for 4 days to ensure that the cells are in the exponential phase by the time of inoculation in 100 mL of Conway medium (Tompkins et al., 1995) containing 1 mL L^–1^ of micronutrients (100 g L^–1^ NaNO_3_, 45 g L^–1^ EDTA, 33.6 g L^–1^ H_3_BO_3_, 20 g L^–1^ NaH_2_PO_4_, 1.30 g L^–1^ FeCL_3_, 0.36 g L^–1^ MnCL_2_), 0.1 mL L^–1^ of trace metals (2.10 g L^–1^ ZnCL_2_, 2 g L^–1^ CoCL_3_, 0.90 g L^–1^ (NH_4_)_6_MO_7_O_2_, 2 g L^–1^ CuSO_4_.5H_2_O), 2 mL of silicate solution (15 g L^–1^ Na_2_SiO_3_) and 0.1 mL L^–1^ of vitamins solution. The pre-inoculum was then inoculated at an initial optical density of 0.001 into 250 mL of fresh medium in 300 mL Erlenmeyer flasks (triplicate). Each algal culture was maintained at 25°C and salinity of 30 ppt under an illumination intensity of 150 μmol m^–2^ s^–1^ under a 12:12 h light/dark photoperiod until the exponential phase. Daily growth was monitored by optical density measurement at 750 nm (OD_750_) using a microplate reader (Varioskan LUX, Thermo, Japan). On day 7, all cells were harvested by centrifugation at 3,500 rpm for 10 min, freeze-dried, and then kept at −20°C before analysis. As a first screening process, the total antioxidant capacity of all strains was spectrophotometrically determined by their scavenging capacity using 2,2’-azino-bis(3-ethylbenzothiazoline-6-sulfonicacid) (ABTS) and 2,2-diphenyl-1-picrylhydrazyl (DPPH) total antioxidant capacity assay (the analytical method has been described in the antioxidant measurement section below).

### Antioxidant Compound Characterization of the Selected Strains

Based on the first screening of their antioxidant capacity, 10 potential microalgal species which produced high antioxidant were selected. The selected species are comprised of bacillariophytes, chlorophytes, and haptophytes. For further experiments involving antioxidant compound characterization, each potential strain was cultivated in triplicate in a 4L Nalgene polycarbonate bottle until the stationary phase, which varied among strains. Aeration was supplied for the whole culture period to provide mixing and equal light and nutrient exposure of the algal cells. The number of days for the exponential and stationary phase of each algal strains was determined based on a preliminary growth curve experiment conducted prior using similar initial cell density and culture conditions. All strains were inoculated at an initial optical density of 0.010, and the growth of each strain was monitored by determining optical density at 750 nm (OD_750_) and dry weight (DW). The optical density was measured daily using a 96-well microplate. Briefly, 200 μL algae sample was added into each well, and OD_750_ was measured using a microplate reader. The total biomass dry weight (g DW L^–1^) of each strain was obtained according to Zhu and Lee (1997) by filtering a known volume of algal suspension using a pre-weighed Whatman GF/F filter, dried for 24 h in an oven and weighed. Using the DW data, the specific growth rate (SGR) for each strain was calculated according to Levasseur et al. (1993). Each algal biomass during both exponential and stationary phases was collected, centrifuged, and freeze-dried to evaluate antioxidant activity, as described in the next section.

### Antioxidant Quantification

The antioxidant capacity was evaluated by a total of four antioxidant assays, including the two radical scavenging assays, ABTS and DPPH, and two other assays that are more specific against the ROS: superoxide radical (O_2_^•–^) scavenging capacity and RNS: Nitric oxide (•NO^–^) scavenging capacity was also analyzed at different growth phases. Details of the measurements are shown below.

First, 2 mL of pure methanol (Wako Chemical, Japan) was added to a tube of 1 mg of finely ground freeze-dried sample and shaken vigorously for 30 s. The sample was sonicated in an ice bath for 45 min (35 kHz, 240 W). The samples were then centrifuged at 5,000 g for 10 min at 4°C and the supernatant was separated. The collected supernatant was combined and used immediately in analysis or purged to dryness using nitrogen gas before it was stored at -20°C.

The total antioxidant radical-scavenging capacities were evaluated using the ABTS radical cation (ABTS^•+^) assay, as originally described by Re et al. (1999). ABTS^•+^ radical cation was prepared by mixing 7 mol m^–3^ ABTS (Wako Chemical, Japan) with 2.45 mol m^–3^ potassium persulfate (K_2_S_8_O_4_, Wako Chemical, Japan) for 8 h in the dark at room temperature (21°C). Briefly, 350 μl of the microalgal extract was added to 1 mL of the ABTS^•+^ solution, and the absorbance was measured at 734 nm using a spectrophotometer (Shimadzu UV-1601, Japan) exactly 1 min after initial mixing and up to 6 min. The results were then expressed as micromole Trolox (TE) equivalent per gram of dry algae biomass (μmol TE g^–1^ DW).

The total antioxidant capacity was also measured using the 1,1-diphenyl-2-picrylhydrazyl (DPPH) stable radical, according to Brand-Williams et al. (1995). The sample extract was added to 3.9 mL of 0.06 mM DPPH radicals (Sigma-Aldrich, MO, United States) in methanol solution and then incubated in the dark for 30 min at room temperature. The reduction of the DPPH free radical was measured at 517 nm using a spectrophotometer. The results were also expressed as μmol TE g^–1^ DW.

Meanwhile, superoxide anions were generated in a phenazine methosulphate-NADH system and assayed by reduction of nitro blue tetrazolium (NBT) as described by Robak and Gryglewski (1988) with slight modifications (Abdullah et al., 2017). All reagents were prepared using 0.1 M phosphate-buffered saline. In a 96-well microplate, 50 μL NBT (150 μM), 50 μL algal extract, and 50 μL NADH (468 μM) were mixed with 50 μL phenazine methosulfate (60 μM). Absorbance was read at 570 nm using a microplate reader after incubation in the dark for 10 min at room temperature. The results were expressed as μmol TE g^–1^ DW.

The nitric oxide radical scavenging activity was determined, according to Oliveira et al. (2010). Briefly, 100 μL of the algal extract was incubated with 100 μL of sodium nitroprusside (20 mM) under light at room temperature. After 60 min of incubation, Griess reagent containing 1% sulphanilamide and 0.1% naphthylethylenediamine in 2% phosphoric acid was added to each well. After 10 min incubation, the absorbance of the chromophore formed during the diazotization of nitrite with sulphanilamide and subsequent coupling with naphthylethylenediamine was read at 562 nm, and the results were expressed as μmol TE g^–1^ DW.

### Antioxidant Compound Profiling

Carotenoids in each extract were tentatively identified and quantified according to using a reverse-phase gradient elution HPLC system, according to Zapata et al. (2000). The algal methanolic extract was filtered through a 0.2 μm PTFE filter (Whatman) before it was injected into the HPLC system (Shimadzu) equipped with a Symmetry C8 column (Waters). The mobile phase comprised of the following two solvents: (A) methanol: acetonitrile: aqueous 0.25 M pyridine solution (50:25:25 v:v:v), (B) acetonitrile: methanol (80:20 v:v). The solvent gradient was as follows: (1) Solvent A 100% for 18 min; (2) to 60% solvent A, 40% solvent B for 12 min, and (3) to 100% solvent B for 10 min. Carotenoids were identified by comparing their elution order and UV-Vis spectra with chromatographic HPLC-grade standards under identical conditions: diadinoxanthin, fucoxanthin, α- and β-carotene, astaxanthin, antheraxanthin, neoxanthin, violaxanthin, zeaxanthin, lutein, diatoxanthin and Chl *a* were estimated from respective peaks calibrated against pure standards (Danish Hydraulic Institute Water and Environment, Denmark).

Total crude lipids were extracted from the freeze-dried samples with a mixture of chloroform: methanol (1:2, v:v) according to Bligh and Dyer (1959), and fatty acid methyl esters were then produced from the lipid extracts by direct transesterification in methanol containing 5% acetyl chloride at 100°Ñ for 1 h. Fatty acid methyl esters (FAME) were analyzed in a gas chromatograph (GC353, GL-Sciences) using a flame ionization detector and an Agilent DB-FFAP column (30 m length, 0.25 mm inner diameter and 0.25 μm film thickness). The oven heating program comprised a linear increase in column temperature from 160 to 240°C at a rate of 4°C min^–1^. EZ Chrom Elite software (ver. 3.1.7J, GL-Sciences) was used for recording and integration. Chromatographic grade standards of fatty acids in methyl ester formed from Nu-Check-Prep (GLC-68D, United States) containing 20 FAME and from Kitasato University containing 13 FAME were used for tentative peak identification.

The total phenolic content was determined using Folin–Ciocalteu reagent described by Singleton and Rossi (1965). One hundred microliters of each methanolic sample extract were mixed with 400 μL of Folin–Ciocalteu reagent and then incubated at room temperature (5 min) before the addition of 500 μL sodium bicarbonate solution (7.5% w/v). After incubation for 90 min in the dark, absorbance was measured at 760 nm using a microplate reader. Gallic acid was used as the standard. The results were then expressed as gallic acid equivalent (GAE) mg g^–1^ dry weight of microalgae.

### Statistical Analysis

The experiments were carried out in triplicate, and all results are expressed as mean ± standard error. Data were then analyzed using one-way variance analysis (ANOVA), followed by Tukey’s *post-hoc* comparison test to measure differences between data. Statistical significance was determined at *p* < 0.05. Correlations among antioxidant capacities and contents of bioactive compounds were calculated using Pearson’s correlation coefficient (r). Statistical analysis was carried out using the statistical software SPPS, version 23 (SPSS Inc., United States).

## Results

### Selection of Indigenous Microalgae With High Antioxidant Capacity and Growth Capacity

The newly isolated indigenous microalgal strains were identified as *Nanochlorum eucaryotum* (two strains), *Picochlorum maculatum* (two strains), *Chlorella sorokiniana* (two strains), *Oocystis heteromucosa*, *Oocystis marina*, and *Chlamydomonas uva-maris* as chlorophytes, and *Amphora montana* (two strains), *Nitzschia capitellata* (two strains), *Nitzschia palea*, *Psammodictyon pustulatum*, *Pauliella taeniata*, *Navicula arenaria* (three strains), *Navicula radiosa* (two strains) and *Thalassiosira weisflogii* (two strains) as bacillariophytes (see Supplementary Table 1).

The 26 indigenous microalgae showed that antioxidant capacity varied among division, class, species, and strain (Table 2). Among the chlorophytes, the highest scavenging effect against ABTS was obtained by *Chlorella sorokiniana* (SLG4-12) at 34.16 ± 1.18 μmol TE g^–1^ DW, followed by *Nanochlorum eucaryotum* (SLG4-08, SLG4-11), and *Chlorella sorokiniana* (SLG4-13), and *Tetraselmis suecica.* On the other hand, the lowest (*p* < 0.05) antioxidant capacity among the chlorophyte strains was observed in *Chlamydomonas uva-maris* (SLG4-14) at 4.32 ± 0.02 μmol TE g^–1^ DW. The only haptophyte strain evaluated was *Isochrysis galbana* (33.60 ± 0.67 μmol TE g^–1^ DW), showed higher (*p* < 0.05) antioxidant capacity by ABTS than the bacillariophyte strain *Phaeodactylum tricornutum* (control) at 25.23 ± 0.12 μmol TE g^–1^ DW. Among the 12 pennate diatoms studied, only *Amphora montana* (SLG4-03) showed higher (*p* < 0.05) antioxidant capacity in ABTS than that of the control strain (25.23 ± 0.12 μmol TE g^–1^ DW) at 30.74 ± 0.05 μmol TE g^–1^ DW. The lowest (*p* < 0.05) ABTS scavenging capacity among all diatoms was observed in *Navicula arenaria* (SLG4-18) at only 5.33 ± 1.14 μmol TE g^–1^ DW, respectively. All three centric diatom strains, *Chaetoceros gracilis*, *Thalassiosira weissflogii* (TRG10-P103), and *Thalassiosira weissflogii* (TRG10-P105), indicated a higher (*p* < 0.05) total antioxidant capacity than *Phaeodactylum tricornutum*.

**TABLE 2:** Antioxidant capacity of tropical microalgal strains from Malaysian waters, assessed by the ABTS and DPPH radical scavenging assay, expressed as micromol equivalent to Trolox per g of biomass dry weight (mean ± SE).

Division	Class	Strain	ABTS (μmol trolox g^–1^ DW)	DPPH (μmol trolox g^–1^ DW)
Chlorophytes (Green algae)	Trebouxiophyceae	*Nanochlorum eucaryotum* SLG4-08	29.08 ± 0.02	8.51 ± 0.28
		*Nanochlorum eucaryotum* SLG4-11	**26.72 ± 1.67**	**9.23 ± 1.45**
		*Picochlorum maculatum* TRG9-05	7.69 ± 0.56	**11.76 ± 1.12**
		*Picochlorum maculatum* TRG9-06	12.71 ± 0.66	5.71 ± 1.80
		*Chlorella sorokiniana* SLG4-12	**34.16 ± 1.18**	**11.29 ± 0.87**
		*Chlorella sorokiniana* SLG4-13	**29.68 ± 0.33**	**8.50 ± 0.22**
		*Chlorella vulgaris*	11.59 ± 1.76	**11.77 ± 0.25**
		*Oocystis heteromucosa* TRG10-P102	7.37 ± 0.37	2.43 ± 1.21
		*Oocystis marina* TRG10-P104	10.45 ± 1.45	1.86 ± 0.02
	Chlorophyceae	*Chlamydomonas uva-maris* SLG4-14	4.32 ± 0.02	5.34 ± 0.08
	Prasinophyceae	*Tetraselmis suecica*	**28.29 ± 0.53**	**12.29 ± 0.29**
Bacillariophytes (Diatom)	Bacillariophyceae (pennate diatom)	*Amphora montana* SLG4-03	**30.74 ± 0.05**	**8.50 ± 0.21**
		*Amphora montana* SLG4-17	13.33 ± 0.18	1.06 ± 0.48
		*Nitzschia capitellata* TRG9-08	15.99 ± 1.87	2.57 ± 0.22
		*Nitzschia capitellata* TRG9-09	7.81 ± 1.48	3.49 ± 2.29
		*Nitzschia palea* SLG4-16	8.66 ± 0.61	6.30 ± 0.20
		*Psammodictyon pustulatum* TRG9-10	10.99 ± 0.94	7.05 ± 0.39
		*Pauliella taeniata* TRG8-02	9.79 ± 0.53	5.24 ± 0.24
		*Navicula arenaria* SLG4-18	5.33 ± 1.14	0.68 ± 0.36
		*Navicula radiosa* TRG9-03	9.67 ± 1.41	6.89 ± 0.61
		*Navicula arenaria* SLG4-01	14.94 ± 0.53	3.28 ± 1.52
		*Navicula radiosa* SLG4-02	10.16 ± 0.41	**8.77 ± 0.43**
		*Phaeodactylum tricornutum*	25.23 ± 0.12	6.68 ± 1.09
	Mediophyceae (centric diatom)	*Thalassiosira weissflogii* TRG10-P103	**27.16 ± 0.02**	**9.38 ± 0.43**
		*Thalassiosira weissflogii* TRG10-P105	**32.49 ± 1.68**	**16.83 ± 1.03**
		*Chaetoceros gracilis*	**30.46 ± 1.16**	**9.56 ± 0.23**
Haptophytes	Coccolithophyceae	*Isochrysis galbana*	**33.60 ± 0.67**	**19.01 ± 1.34**

*Significantly high value among strains are printed in bold (p < 0.05).*

Meanwhile, the total antioxidant capacity, according to the DPPH assay, varied from 0.68 ± 0.36 to 19.01 ± 1.34 μmol TE g^–1^ DW (Table 2). The bacillariophytes and haptophytes with significantly higher (*p* < 0.05) antioxidant capacity than the control (6.68 ± 1.09 μmol TE g^–1^ DW) were *Isochrysis galbana* (19.01 ± 1.34 μmol TE g^–1^ DW), *Thalassiosira weissflogii* (TRG10-P105) (16.83 ± 1.03 μmol TE g^–1^ DW), *Chaetoceros gracilis* (9.56 ± 0.23 μmol TE g^–1^ DW), *Amphora montana* (SLG4-03) (8.50 ± 0.21 μmol TE g^–1^ DW) and *Navicula radiosa* (SLG4-02) (6.89 ± 0.61 μmol TE g^–1^ DW), respectively. Among the chlorophytes, high (*p* < 0.05) DPPH scavenging capacity compared to control was observed in *Tetraselmis suecica*, *Chlorella vulgaris*, *Chlorella sorokiniana* (SLG4-12), *Nanochlorum eucaryotum* (SLG4-11), *Chlorella sorokiniana* (SLG4-13), and *Nanochlorum eucaryotum* (SLG4-08) ranging from 12.29 ± 0.29 to 8.51 ± 0.28 μmol TE g^–1^ DW, respectively.

Based on the screening results (Table 2), 10 strains were selected for their high antioxidant production and were subjected to further analysis. These include 1. Chlorophyte strains; *Nanochlorum eucaryotum* (SLG4-08), *Nanochlorum eucaryotum* (SLG4-11), *Chlorella sorokiniana* (SLG4-12), *Chlorella sorokiniana* (SLG4-13), and *Tetraselmis suecica*, 2. Bacillariophyte strains; *Amphora montana* (SLG4-03), *Chaetoceros gracilis*, *Thalassiosira weissflogii* (TRG10-P103) and *Thalassiosira weissflogii* (TRG10-P105), and 3. Haptophyte strain; *Isochrysis galbana*.

### Growth Characteristics of Selected Microalgae Candidate Strain at Different Growth Phases

**FIGURE 1:**
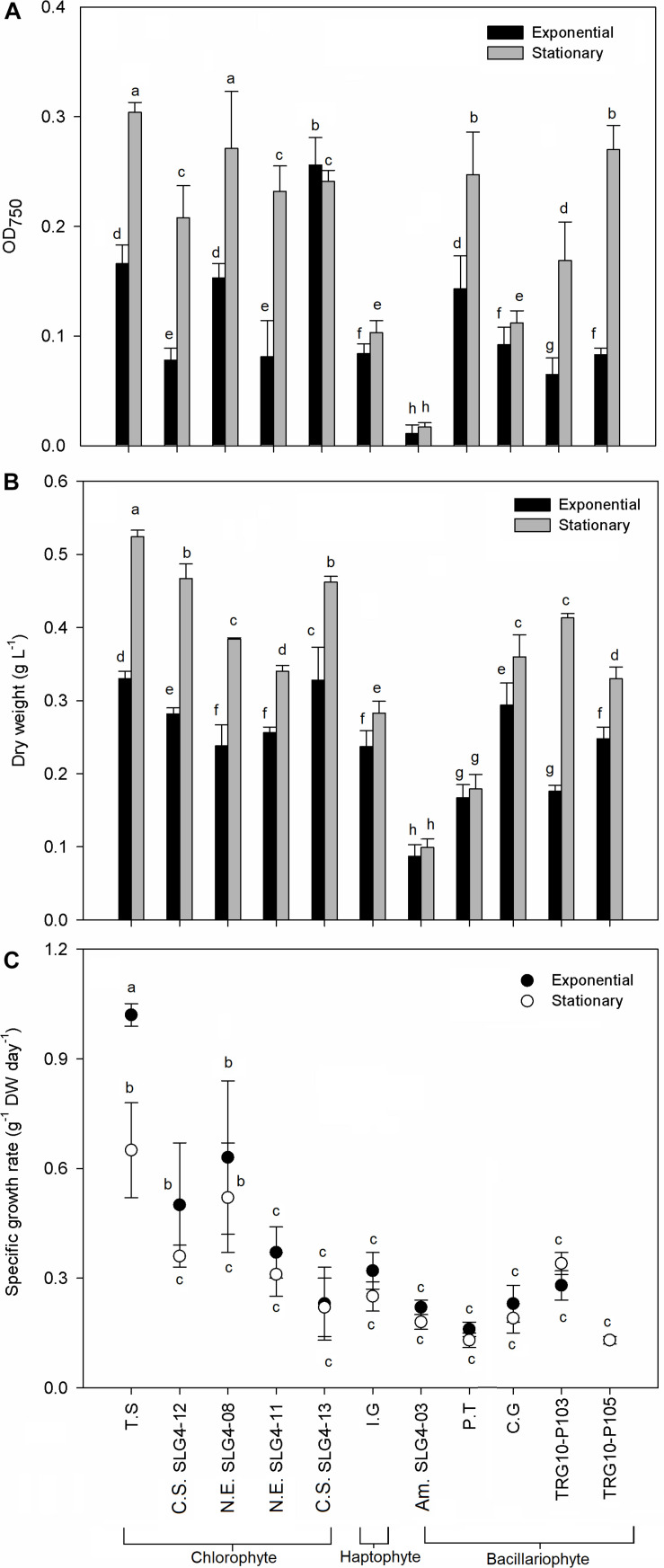
Growth characteristic by **(A)** optical density, **(B)** dry weight, and **(C)** dry weight specific growth rate of selected microalgae strains at exponential and stationary phase. Means denoted by a different letter indicate significant differences between strains (*p* < 0.05); Pico SLG4-12, SLG4-11, SLG4-08, *Picochlorum* sp.; T.S, *Tetraselmis suecica*; C.S. SLG4-13, *Chlorella sorokiniana*; I.G, *I. galbana*; Am. SLG4-03, *Amphora* sp.; P.T, *Phaeodactylum tricornutum*; C.G, *Chaetoceros gracilis*; TRG10-P103, *Thalassiosira* sp.; TRG10-P105, *Thalassiosira weissflogii*.

Growth characteristics in terms of maximum optical density, maximum dry biomass weight, and specific growth rate were different among the 10 selected potential strains as natural antioxidant producers (Figure 1). Under stationary phases, the highest (*p* < 0.05) SGR, OD_750_ and biomass dry weight among all strains were found in the chlorophyte *Tetraselmis suecica* at 1.420 ± 0.31 g^–1^ DW day^–1^, 0.304 ± 0.009 OD_750_ and 0.524 ± 0.002 g DW L^–1^. Meanwhile, under the exponential phase, the highest (*p* < 0.05) OD_750_ and biomass dry weight was observed in the *Chlorella sorokiniana* (SLG4-13) at 0.256 ± 0.025 OD_750_ and 0.328 ± 0.045 g DW L^–1^, respectively. Bacillariophyte, chlorophyte, and haptophyte strains showed relatively higher OD_750_ and biomass dry weight during the stationary phase compared to that during the exponential phase.

### Carotenoid Composition at Different Growth Phases

**FIGURE 2:**
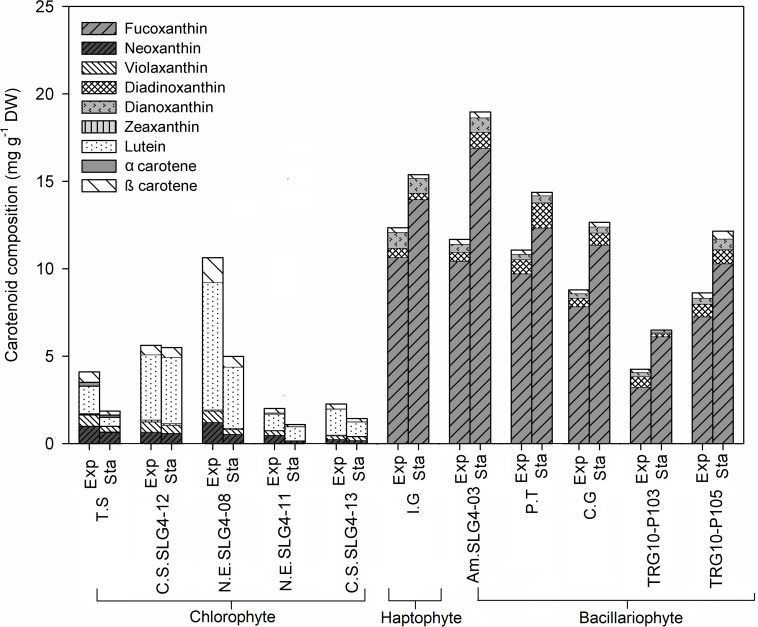
Carotenoid content and composition (mg g^–1^ DW) of selected microalgae strains at exponential (Exp) and stationary phase (Sta). Abbreviations of the strains as for Figure 1.

The microalgal biomass showed division and species-specific carotenoid profiles (Figure 2). Generally, total carotenoids in bacillariophytes and haptophytes are higher than those in chlorophytes. Total carotenoids in bacillariophytes and haptophytes were consistently higher in the stationary phase than in the exponential phase and could be characterized by the presence of fucoxanthin, diadinoxanthin, and diatoxanthin. In particular, the higher total carotenoid content of bacillariophytes and the haptophyte *Isochrysis galbana* compared to chlorophytes was mainly due to their high fucoxanthin composition. The highest (*p* < 0.05) total carotenoid in the stationary phase was observed in *Amphora montana* (SLG4-03), followed by *Isochrysis galbana* and the control *Phaeodactylum tricornutum* at 18.97 ± 3.12, 15.66 ± 2.13, and 14.37 ± 1.84 mg g^–1^ DW, respectively. Correspondingly, the highest fucoxanthin level under the stationary phase was also observed in *Amphora montana* (SLG4-03) (16.90 ± 2.75 mg g^–1^ DW) followed by *Isochrysis galbana*, *Phaeodactylum tricornutum*, *Chaetoceros gracilis*, and *Thalassiosira weissflogii* (TRG10-P105) at 13.96 ± 1.91, 12.34 ± 1.65, 11.37 ± 2.11, and 10.29 ± 0.17 mg g^–1^ DW, respectively. Dry weight-specific fucoxanthin content was generally higher during the early stationary phase than in the exponential phase. Meanwhile, total carotenoids of chlorophytes tended to be higher in the exponential phase than in the early stationary phase and can be characterized by the presence of lutein, neoxanthin, violaxanthin, and zeaxanthin. Total carotenoid content (10.68 ± 4.09) was higher in the chlorophyte strain *Nanochlorum eucaryotum* (SLG4-08), in the exponential phase, as compared to the other chlorophyte strains with relatively rich contents of β-carotene, neoxanthin, violaxanthin, and zeaxanthin.

### Fatty Acid Composition at Different Growth Phases

**FIGURE 3:**
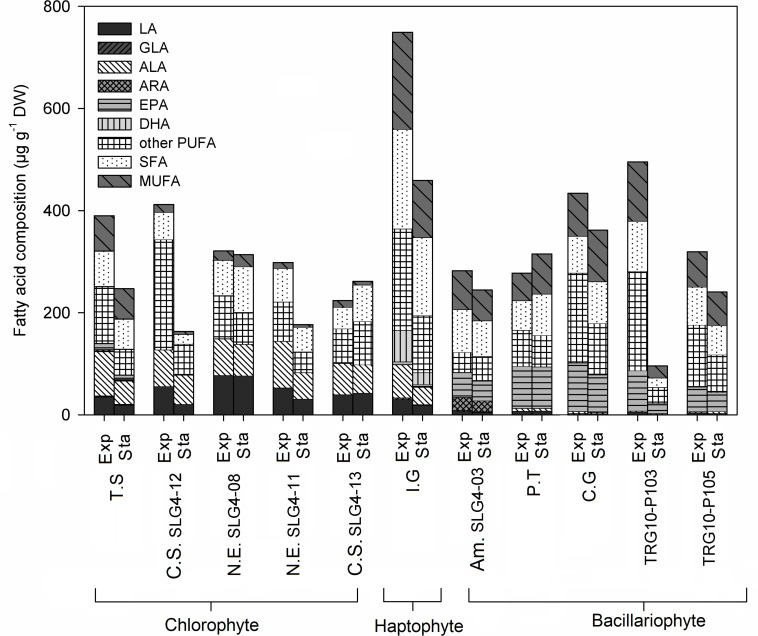
Fatty acid (FA) composition (μg g^–1^ DW) of selected microalgae strains at exponential (Exp) and stationary (Sta) phase. Abbreviations of the strains as for Figure 1. ALA, α- linolenic acid; LA, Linoleic acid; ARA, Arachidonic acid; EPA, Eicosapentaenoic acid; GLA, γ-Linolenic acid; DHA, Docosahexaenoic acid; PUFA, Polyunsaturated fatty acid; SFA, Saturated fatty acid; MUFA, Monounsaturated fatty acid.

The highest fatty acid content was found in *Isochrysis galbana* under exponential phase at 749.25 μg g^–1^ DW, followed by *Thalassiosira weissflogii* (TRG10-P103) (495.46 ± 60.32 μg g^–1^ DW: exponential phase) and *Chaetoceros gracilis* (433.64 ± 220.34 μg g^–1^ DW: exponential phase) (Figure 3). Fatty acids with various degrees of saturation were detected, including saturated (SFA) and monounsaturated and polyunsaturated fatty acids (MUFA, PUFA), while their relative importance was differed depending on the strain. *Isochrysis galbana* and *Chlorella sorokiniana* (SLG4-12) contained the highest amount of PUFA compared to other strains during the exponential phase at 364.33 ± 73.43 and 343.45 ± 52.69 μg g^–1^ DW, respectively. High PUFA composition observed in chlorophytes is contributed mainly by their α-linoleic acid (ALA) and linoleic acid (LA) content. Whereas, for bacillariophytes, PUFA was mainly contributed by their eicosapentaenoic acid (EPA) content. Among the bacillariophytes, *Chaetoceros gracilis, Phaeodactylum tricornutum*, and *Thalassiosira weissflogii* (TRG10-P103), the high EPA content was observed during the exponential phase at 94.93 ± 39.89, 82.29 ± 8.34, and 78.62 ± 1.06 μg g^–1^ DW, respectively.

Meanwhile, the highest ALA was observed during the exponential phase in *Nanochlorum eucaryotum* (SLG4-11) (91.02 ± 6.26 μg g^–1^ DW), followed by *Tetraselmis suecica* (87.38 ± 15.17 μg g^–1^ DW). Likewise, *Nanochlorum eucaryotum* (SLG4-08) under the exponential phase showed the highest LA content at 77.21 ± 7.08 μg g^–1^ DW compared to other strains. Among the 10 strains analyzed, only *Isochrysis galbana* contained docosahexaenoic acid (DHA) at 61.76 ± 9.37 μg g^–1^ DW during the exponential phase and 23.00 ± 4.30 μg g^–1^ DW during the stationary phase, respectively. Meanwhile, γ-linolenic acid (GLA) was only detected at low concentrations in *Tetraselmis suecica* (exponential: 1.35 ± 0.20; stationary phase: 0.95 ± 0.16 μg g^–1^ DW), *Isochrysis galbana* (exponential: 3.81 ± 1.27; stationary phase: 1.47 ± 0.69 μg g^–1^ DW), *Amphora montana* (SLG4-03) (exponential: 2.34 ± 0.83; stationary phase: 1.28 ± 0.88 μg g^–1^ DW), and *Chaetoceros gracilis* (exponential:1.81 ± 1.04; stationary phase 2.23 ± 0.63 μg g^–1^ DW). The highest ARA was observed in *Amphora montana* (SLG4-03) at 25.08 ± 7.06 μg g^–1^ DW at the exponential phase.

### Total Phenolic Content at Different Growth Phases

The phenolic content of the 10 strains ranged from 0.53 to 15.54 mg GAE g^–1^ DW under the exponential phase and from 0.79 to 11.80 mg GAE g^–1^ DW under the stationary phase (Table 3). The phenolic content varied among species, although generally higher content was observed in the exponential phase, except for *Amphora montana* (SLG4-03) and *Phaeodactylum tricornutum* (Table 3). The highest phenolic content was found under the exponential phase in *Tetraselmis suecica* at 15.54 ± 2.22 mg GAE g^–1^ DW, followed by *Isochrysis galbana* and *Thalassiosira weissflogii* (TRG10-P105) at 14.53 ± 2.04 and 12.46 ± 1.55 mg GAE g^–1^ DW, respectively (Table 3).

**TABLE 3:** Total phenolic content in the selected microalgae biomass for antioxidant compound production.

Division	Class	Strain	Total phenolic (mg GAE g^–1^ DW)	
			Exponential phase	Stationary phase
Chlorophytes (Green algae)	Trebouxiophyceae	*Nanochlorum eucaryotum* SLG4-08	**6.80 ± 1.68**	0.79 ± 0.11
		*Nanochlorum eucaryotum* SLG4-11	**7.27 ± 1.41**	5.97 ± 1.03
		*Chlorella sorokiniana* SLG4-12	**10.17 ± 1.44**	6.79 ± 1.76
		*Chlorella sorokiniana* SLG4-13	**5.34 ± 1.26**	3.11 ± 1.08
	Prasinophyceae	*Tetraselmis suecica*	**15.54 ± 2.22**	11.80 ± 0.60
Bacillariophytes (Diatom)	Bacillariophyceae (pennate diatom)	*Amphora montana* SLG4-03	0.53 ± 1.03	**5.97 ± 1.05**
		*Phaeodoctylum tricornutum*	10.64 ± 1.03	11.09 ± 1.55
	Mediophyceae (centric diatom)	*Thalassiosira weissflogii* TRG10-P103	**7.04 ± 1.67**	2.32 ± 1.82
		*Thalassiosira weisflogii* TRG10-P105	**12.46 ± 1.55**	9.38 ± 2.41
		*Chaetoceros gracilis*	**10.97 ± 1.21**	4.50 ± 1.24
Haptophytes	Coccolithophyceae	*Isochrysis galbana*	**14.53 ± 2.04**	7.32 ± 0.27

*Significantly high value among growth phases are printed in bold (p < 0.05).*

### Interrelationship of Microalgae Antioxidant Capacity With Carotenoid, Fatty Acid, and Phenolic Contents at Different Growth Phases

**FIGURE 4:**
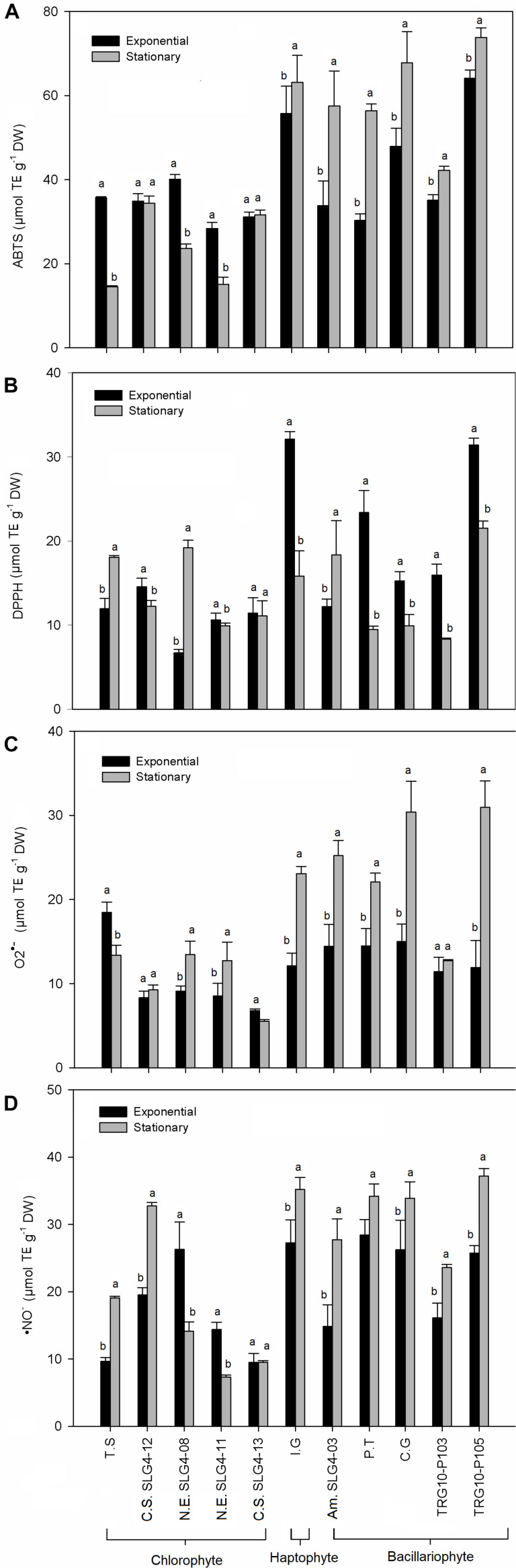
Antioxidant activity obtained for the total antioxidant capacity and biological radical scavenging assay performed **(A)** ABTS^•+^, **(B)** DPPH•, **(C)** O_2_^•–^, and **(D)** NO*^–^*. Means denoted by a different letter indicate significant differences between growth phase (*p* < 0.05); Abbreviations of the strains as for Figure 1.

The total antioxidant capacity (by ABTS and DPPH assays) showed a group-specific variation (Figures 4A,B). Higher (*p* > 0.05) total antioxidant capacity among the bacillariophyte and haptophyte strains compared to chlorophyte was found in *Thalassiosira weissflogii* (TRG10-P105) with ABTS and DPPH scavenging at 73.8 ± 1.94 (stationary phase) and 31.42 ± 0.80 (exponential phase) μmol TE g^–1^ DW, respectively. ABTS scavenging activity was also high in *Chaetoceros gracilis* (67.79 ± 7.45 μmol TE g^–1^ DW) and *Isochrysis galbana* (63.12 ± 6.51 μmol TE g^–1^ DW) during the stationary phase compared to the chlorophyte strains. Meanwhile, among the chlorophyte strains, the highest (*p* > 0.05) total antioxidant capacity was found in *Nanochlorum eucaryotum* (SLG4-08) under the exponential phase with ABTS scavenging at 40.11 ± 1.11 μmol TE g^–1^ DW. As for DPPH, *Isochrysis galbana* at the exponential phase showed a scavenging capacity at 32.11 ± 0.89 μmol TE g^–1^ DW. The highest O_2_^•–^ scavenging capacity was observed in *Chaetoceros gracilis* and *Thalassiosira weissflogii* (TRG10-P105) under the early stationary phase at 30.4 ± 3.66 and 30.94 ± 3.15 μmol TE g^–1^ DW, respectively (Figure 4C). Similarly, the highest NO*^–^* scavenging capacity was also observed in both strains during the early stationary phase (Figure 4D).

The results of the Parsons correlation indicated that carotenoids and phenolic compounds were significant contributors to the antioxidant scavenging capacity of the microalgae methanol extracts (Table 4). Meanwhile, a low influence with antioxidant capacity was observed for total fatty acid and PUFA compared to total carotenoid and total phenolic content. The variation in the antioxidant capacities of the bacillariophytes, chlorophytes, and haptophytes under different growth phases was closely related to the differences in the production of the bioactive compounds, especially carotenoids and phenolic compounds. In particular, for the selected bacillariophyte and haptophyte strains, the relatively higher *R*^2^-values of the correlation analysis for ABTS (0.623), DPPH (0.714), and •NO^–^ (0.786) compared to total carotenoid, total fatty acid, and PUFA, suggest that their antioxidant capacity was significantly influenced by the phenolic content during the exponential phase (*p* < 0.05). Meanwhile, during the stationary phase, their antioxidant capacity was significantly contributed by carotenoids, as observed with ABTS (0.565), •NO^–^ (0.466), and O_2_^•–^ (0.657). As for the chlorophyte strains, significant correlations with antioxidant capacity were observed in carotenoids (ABTS and •NO^–^) and phenolic (•NO^–^ and O_2_^•–^) during both the exponential and stationary phases (Table 4).

**TABLE 4:** Parsons correlation, between antioxidant capacity and total carotenoid, total phenolic, total fatty acid (FAs), and total polyunsaturated fatty acid (PUFAs) in different algal group analyzed in this study.

Division	Growth stage	Antioxidant capacity	Total carotenoid	Total phenolic	Total FAs	Total PUFA
Bacillariophytes (Diatom) and haptophyte*	Exponential	ABTS	0.104	**0.623**	0.350	−0.142
		DPPH	0.275	**0.714**	0.324	0.188
		•NO^–^	0.141	**0.786**	0.141	0.403
		O_2_^•–^	0.267	−0.255	0.267	0.069
	Stationary	ABTS	**0.565**	−0.027	0.117	0.064
		DPPH	−0.225	0.104	−0.074	−0.129
		•NO^–^	**0.466**	0.297	0.149	0.072
		O_2_^•–^	**0.657**	0.082	0.175	0.121
Chlorophytes (Green algae)	Exponential	ABTS	**0.591**	0.023	0.486	0.399
		DPPH	−0.081	0.089	0.243	0.356
		•NO^–^	**0.655**	−0.271	0.027	0.168
		O_2_^•–^	−0.222	**0.669**	0.437	0.173
	Stationary	ABTS	**0.517**	−0.283	−0.227	0.030
		DPPH	0.350	0.125	0.441	0.256
		•NO^–^	**0.642**	**0.505**	−0.226	−0.148
		O_2_^•–^	0.135	0.168	−0.080	−0.260

Significant correlations among growth phases are printed in bold (p < 0.05), O_2_^•–^, Superoxide radical scavenging capacity; •NO^–^, Nitric oxide radical scavenging capacity. *Haptophyte (Isochrysis galbana) was analyzed with strains belonging to Bacillariophytes due to its similarity in carotenoid composition (see Figure 2).

Detailed analysis of the correlation with each carotenoid showed that their antioxidant capacity (ABTS and O_2_^•–^) was significantly (*p* < 0.05) influenced by their fucoxanthin, diadinoxanthin, and β-carotene content for bacillariophytes and haptophytes (Table 5). For the chlorophyte strains during the exponential phase, ABTS showed a significant correlation with β-carotene (*R*^2^-value = 0.615), lutein (0.615), and zeaxanthin (0.507). Similarly, •NO^–^ was also largely contributed by lutein (0.794), β-carotene (0.659), and neoxanthin (0.509). Meanwhile, a significant influence of α-carotene was observed for O_2_^•–^ (0.534). In the stationary phase, chlorophytes were significantly influenced (*p* < 0.05) by the presence of violaxanthin (0.755 with •NO^–^), neoxanthin (0.623 with DPPH, 0.610 with •NO^–^), and lutein (0.582 with •NO^–^).

**TABLE 5:** Parsons correlation between antioxidant capacity and each carotenoid compounds in different algal group analyzed in this study.

Division	Growth stage	Antioxidant capacity	Fuco	Diadino	Diato	β-caro	Neo	Viola	Zea	Lutein	α-caro
Bacillariophytes	Exponential	ABTS	**0.530**	0.220	0.184	**0.581**	–	−	−	−	−
(Diatom) and		DPPH	0.107	0.103	0.071	0.351	−	−	−	−	−
haptophyte*		•NO^–^	0.179	0.192	0.236	0.193	−	−	−	−	−
		O_2_^•–^	−0.093	−0.112	−0.311	−0.584	−	−	−	−	−
	Stationary	ABTS	**0.609**	**0.472**	0.372	**0.689**	−	−	−	−	−
		DPPH	−0.211	−0.338	−0.229	−0.046	−	−	−	−	−
		•NO^–^	0.405	0.492	0.291	0.408	−	−	−	−	−
		O_2_^•–^	**0.710**	**0.631**	0.523	**0.795**	−	−	−	−	−
Chlorophytes	Exponential	ABTS	–	−	−	**0.615**	0.434	0.466	**0.507**	**0.615**	−0.060
(Green algae)		DPPH	−	−	−	−0.400	−0.040	0.059	0.300	−0.374	0.304
		•NO^–^	−	−	−	**0.695**	**0.509**	0.412	0.270	**0.794**	−0.182
		O_2_^•–^	−	−	−	0.146	0.373	0.475	0.190	−0.087	**0.534**
	Stationary	ABTS	−	−	−	0.390	0.108	0.446	0.421	0.487	−0.423
		DPPH	−	−	−	0.403	**0.623**	0.339	0.210	0.263	0.456
		•NO^–^	−	−	−	0.499	**0.610**	**0.755**	0.483	**0.582**	0.065
		O_2_^•–^	−	−	−	0.178	0.387	−0.006	−0.242	0.099	0.342

Significant correlations among growth phases are printed in bold (p < 0.05). Fuco, Fucoxanthin; Diadino, Diadinoxanthin; Diato, Diatoxanthin; β-caro, β carotene; Neo, Neoxanthin; Viola, Violaxanthin; Zea, Zeaxanthin; α-caro, α carotene; O_2_^•–^, Superoxide radical scavenging capacity; •NO^–^, Nitric oxide radical scavenging capacity. *Haptophyte (Isochrysis galbana) was analyzed with strains belonging to Bacillariophytes due to its similarity in carotenoid composition (see Figure 2). -: not detected.

Some essential fatty acids also showed a significant correlation with antioxidant capacity, especially during the exponential phase. For bacillariophytes and haptophytes (Table 6), the contribution of LA (R^2^ value = 0.559), ALA (0.510), and DHA (0.536) toward antioxidant capacity was observed for DPPH, while they were lower than the R^2^ value of total phenolic (0.714) (Table 4). No significant relationships with essential fatty acids were found in the stationary phase of bacillariophytes and haptophytes strains. Meanwhile, for the chlorophytes, the highest influence on antioxidant capacity (O_2_^•–^) was observed for ARA (0.923) and EPA (0.947). In addition, LA also showed a significant contribution toward ABTS (0.566) and •NO^–^(0.781). Significant relationships were also observed under the stationary phases of the chlorophytes for DPPH with ARA (0.503) and EPA (0.517).

**TABLE 6:** Parsons correlation between antioxidant capacity and each essential fatty acid compounds in different algal group analyzed in this study.

Division	Growth stage	Antioxidant capacity	LA (C18:2 n-6)	GLA (C18:3 n-6)	ALA (C18:3 n-3)	ARA (C20:4 n-6)	EPA (C20:5 n-3)	DHA (C22:6 n-3)	Total PUFA	Total fatty acid
Bacillariophytes	Exponential	ABTS	0.332	−0.318	0.324	−0.100	−0.404	0.356	−0.142	0.350
(Diatom) and		DPPH	**0.559**	−0.142	**0.510**	−0.284	−0.349	**0.536**	0.188	0.324
haptophyte*		•NO^–^	0.343	0.173	0.361	−0.440	−0.024	0.350	0.403	0.141
		O_2_^•–^	−0.306	−0.018	−0.207	0.030	0.318	−0.202	0.069	0.267
	Stationary	ABTS	−0.041	0.048	−0.062	0.187	0.321	−0.106	0.064	0.117
		DPPH	0.034	−0.336	−0.103	0.188	−0.050	−0.106	−0.129	−0.074
		•NO^–^	0.174	−0.193	0.186	−0.126	0.021	0.244	0.072	0.149
		O_2_^•–^	0.033	−0.057	−0.018	0.301	0.336	0.006	0.121	0.175
Chlorophytes	Exponential	ABTS	**0.566**	−0.18	0.145	0.227	0.218	–	0.399	0.458
(Green algae)		DPPH	−0.538	0.015	**0.517**	0.036	0.135	–	0.356	0.243
		•NO^–^	**0.781**	−0.251	0.056	−0.457	−0.487	–	0.168	0.027
		O_2_^•–^	−0.357	−0.242	0.068	**0.923**	**0.947**	–	0.173	0.437
	Stationary	ABTS	−0.027	−0.053	0.019	−0.507	−0.545	–	0.03	−0.227
		DPPH	0.352	0.310	0.047	**0.503**	**0.517**	–	0.256	0.441
		•NO^–^	−0.341	−0.076	0.021	0.117	0.112	–	−0.148	−0.226
		O_2_^•–^	−0.045	0.349	−0.206	0.347	0.318	–	−0.026	−0.08

Significant correlations among growth phases are printed in bold (p < 0.05). ALA, α- linolenic acid; LA, Linoleic acid; ARA, Arachidonic acid; EPA, Eicosapentaenoic acid; GLA, γ-Linolenic acid; DHA, Docosahexaenoic acid; O_2_^•–^, Superoxide radical scavenging capacity; •NO^–^, Nitric oxide radical scavenging capacity. *Haptophyte (Isochrysis galbana) was analyzed with strains belonging to Bacillariophytes due to its similarity in fatty acid composition (see Figure 3). –: not detected.

## Discussion

In the present study, 26 indigenous tropical marine tropical microalgae from Malaysian waters were screened for their antioxidant capacity by ABTS and DPPH radical scavenging assays. Both assays measure the total antioxidant capacity through HAT and SET mechanisms of the polar and non-polar compounds present in the extract (Prior et al., 2005). Due to the varying nature and polarity of these compounds, the choice of solvents used during extraction determines its composition and effect on the biological activity. Methanolic solvent has been widely known to have an affinity for a wide range of bioactive compounds, mainly carotenoids and phenolics, as well as high antioxidative properties than extract obtained from other common solvents (Pereira et al., 2015; Safafar et al., 2015). In addition, the different antioxidant molecules in the microalgal methanolic extracts may also act through different mechanisms, which highlights the importance of using at least two assays for the evaluation of antioxidant capacity in natural extracts (Gülçin et al., 2011; Assunção et al., 2017). The results from the methanolic algal extract have often shown considerable variation even between species of the same genus or among multiple isolates of the same species (Safafar et al., 2015). Thus, when dealing with a large number of samples available with the nature of the compounds present in those extracts is unknown, screening by evaluating the antioxidant capacity of the entire extract adopted in this study is more appropriate.

DPPH scavenging assay has been described as less sensitive for samples with high carotenoids, but more sensitive with high phenolic sample (Amaro et al., 2015; Assunção et al., 2017). Meanwhile, ABTS assay is considered as highly sensitive on both carotenoid and phenolic rich sample in the measurement of antioxidant scavenging capacity (Safafar et al., 2015; Assunção et al., 2017). Thus, candidate strain selection was based on strain showing both higher ABTS and DPPH than the control. These selection criteria allowed a more consistent confirmation of the high antioxidant capacity species/strain selection. Based on overall comparisons of the results from the first screening, 10 indigenous tropical microalgal strains comprising several chlorophytes, bacillariophytes, and haptophytes strains were selected as potential candidates showing high total antioxidant capacity. The selected strains were *Nanochlorum eucaryotum* (SLG4-08), *Nanochlorum eucaryotum* (SLG4-11), *Chlorella sorokiniana* (SLG4-12), *Chlorella sorokiniana* (SLG4-13), *Tetraselmis suecica*, *Amphora montana* (SLG4-03), *Chaetoceros gracilis*, *Thalassiosira weissflogii* (TRG10-P103), *Thalassiosira weisflogii* (TRG10-P105) and *Isochrysis galbana*. Among them, eight strains, except for *Chaetoceros gracilis* and *Isochrysis galbana*, which were previously isolated by Natrah et al. (2007), were newly established in this study, indicating that microalgal flora in Malaysian water bodies is a highly promising habitat for antioxidant producers. It was found that several strains, especially *Amphora montana* (SLG4-03), *Thalassiosira weisflogii* (TRG10-P105), *Chaetoceros gracilis*, and *Isochrysis galbana*, were two to three times better in scavenging ABTS radical in comparison to the value reported by Li et al. (2007); Goiris et al. (2012), Safafar et al. (2015), and Agregán et al. (2018) as shown in Table 7. In addition, the carotenoid content in most selected strains in this study showed higher value compared to that in the previously reported algal strains (Table 7). This includes *Isochrysis galbana* with 15.66 ± 2.13 mg g^–1^ DW carotenoid content compared to 4.33 ± 0.04 mg g^–1^ DW and 7.75 ± 0.13 mg g^–1^ DW as reported by Goiris et al. (2012) and Foo et al. (2017). *Chaetoceros gracilis* also showed 12.66 ± 2.45 mg g^–1^ DW of total carotenoid compared to the previously reported *Chaetoceros calcitrans* at 2.33 ± 0.14 (Goh et al., 2010) and 6.13 ± 0.25 mg g^–1^ DW (Foo et al., 2017), respectively. Some other selected species with high total carotenoid content compared to the previously reported one also includes the *Nanochlorum eucaryotum* (SLG4-08), *Amphora montana* (SLG4-03) and *Thalassiosira weissflogii* (TRG10-P105) as shown in Table 7. Our data also showed that bacillariophytes and haptophytes tended to show higher antioxidant capacities than chlorophytes. Although there are previous studies on the screening of antioxidants among different microalgae (Natrah et al., 2007; Goiris et al., 2012; Lauritano et al., 2016; Morowvat and Ghasemi, 2016; Assunção et al., 2017), this is the first thorough comparison across different divisions among the phyla Bacillariophyta, Chlorophyta and Haptophyta, for their antioxidant capacities and bioactive compositions, using the same experimental protocol. This is also the first study to compare the effects of compounds on antioxidants across the three divisions under different growth phases using a standardized unit for better comparison. These results clearly show that the variations in the antioxidant compounds and growth phase influence the antioxidant capacity across the taxonomical group (i.e., fucoxanthin in Bacillariophyta and lutein in Chlorophyta). The effect of the growth phase is mainly due to the nutrient availability factor, which affects the composition of the antioxidant compounds and, thus, the total antioxidant capacity of each algal strains, as we have discussed in detail below.

**TABLE 7:** Comparison of antioxidant capacity and bioactive compounds in high potential Bacillariophyte, Haptophyte, and Chlorophyte strains.

Division	Microalgae strain	Antioxidant capacity		Carotenoid (mg g^–1^ DW)	Phenolic (mg GAE g^–1^ DW)	Growth phase	References
		ABTS (μmol TE g^–1^ DW)	DPPH (μmol TE g^–1^ DW)				
Chloro-phytes	*Nanochlorum eucaryotum* SLG4-08	40.11 ± 1.11	6.73 ± 0.39	10.68 ± 4.09	6.80 ± 1.68	Exp	This study
		23.60 ± 1.10	19.21 ± 0.90	5.01 ± 1.39	0.79 ± 0.11	Sta	
	*Chlorella sorokiniana* SLG4-12	34.88 ± 1.70	14.58 ± 0.97	5.70 ± 0.13	10.17 ± 1.44	Exp	
		34.3 ± 1.72	12.24 ± 0.70	5.59 ± 0.55	6.79 ± 1.76	Sta	
	*Chlorella sorokiniana* SLG4-13	31.13 ± 1.22	11.45 ± 1.79	2.27 ± 0.67	5.34 ± 1.26	Exp	
		31.62 ± 1.22	11.11 ± 1.60	1.45 ± 0.26	3.11 ± 1.08	Sta	
	*Tetraselmis suecica*	35.7 ± 0.27	11.96 ± 1.23	3.94 ± 0.63	15.54 ± 2.22	Exp	
		14.5 ± 0.30	18.09 ± 0.19	1.76 ± 0.24	11.80 ± 0.60	Sta	
	*Nannochloropsis* sp.	–	23.18 ± 0.55	–	–	Sta	Goh et al., 2010
	*Scenedesmus* sp. ME02*	–	3.71	0.61 ± 0.05	5.40 ± 0.28	Exp	Bulut et al., 2019
	Chlamydomonas nivalis**	24.13 ± 0.47	–	–	15.07 ± 0.26	nd	Li et al., 2007
	*Desmodesmus* sp.	24.26 ± 0.60	*29.11%	6.70 ± 0.01	7.72 ± 0.08	Sta	Safafar et al., 2015
	Scenedesmus rubescens**	–	68.68 ± 5.95	–	48.57 ± 3.99	Sta	Morowvat and Ghasemi, 2016
	Chlorella vulgaris*	15.64	0.86	–	–	nd	Agregán et al., 2018
Bacillario-phytes	*Amphora montana* SLG4-03	33.79 ± 5.91	12.20 ± 0.94	11.66 ± 3.17	0.53 ± 1.03	Exp	This study
		57.46 ± 8.27	18.34 ± 4.09	18.97 ± 3.12	5.97 ± 1.05	Sta	
	*Thalassiosira weissflogii* TRG10-P103	35.10 ± 1.31	15.94 ± 1.26	7.14 ± 2.24	7.04 ± 1.67	Exp	
		42.18 ± 1.00	8.33 ± 0.15	3.60 ± 0.49	2.32 ± 1.82	Sta	
	*Thalassiosira weisflogii* TRG10-P105	64.12 ± 1.94	31.42 ± 0.14	8.63 ± 1.04	12.46 ± 1.55	Exp	
		73.78 ± 2.31	21.53 ± 0.84	12.15 ± 0.12	9.38 ± 2.41	Sta	
	*Chaetoceros gracilis*	47.88 ± 4.35	15.24 ± 2.31	8.80 ± 4.43	10.97 ± 1.21	Exp	
		67.79 ± 7.45	9.92 ± 0.79	12.66 ± 2.45	4.50 ± 1.24	Sta	
	*Chaetoceros calcitrans*	0.068	–	6.13 ± 0.25	12.24 ± 1.61	nd	Foo et al., 2017
	*Chaetoceros* sp.	–	39.22 ± 2.52	–	–	Sta	Goh et al., 2010
	Chaetoceros calcitrans*	24.24 ± 1.64	–	2.33 ± 0.14	1.84 ± 0.11	nd	Goiris et al., 2012
Hapto-phytes	*Isochrysis galbana*	55.68 ± 6.51	32.11 ± 0.89	12.08 ± 1.80	14.53 ± 2.04	Exp	This study
		63.12 ± 6.51	15.83 ± 3.00	15.66 ± 2.13	7.32 ± 0.27	Sta	This study
	*Isochrysis galbana*	21.55 ± 1.58	–	4.33 ± 0.04	3.68 ± 0.39	nd	Foo et al., 2017
	*Isochrysis* sp.*	22.50 ± 0.93	–	7.75 ± 0.13	4.57 ± 0.18	Exp	Goiris et al., 2012

All data were from algal methanolic extract, otherwise noted. Exp, Exponential phase; Sta, Stationary phase. *Ethanol/water mixture extract. **Hexane/ethyl acetate/water sequential extract. –: not determined.

Our results showed that carotenoids contribute significantly to the antioxidant properties of the selected potential strains. A similar contribution has been reported in previous studies (Goiris et al., 2012; Safafar et al., 2015). It is known that although carotenoids play an important role in quenching reactive oxygen species, different carotenoid compounds exhibit different antioxidative power or action mechanisms (Young and Lowe, 2018). Each carotenoid antioxidant activity depends on the presence of the electron-rich conjugated system of the polyene and functional cyclic end groups, which enables them to scavenge harmful radicals either by electron transfer, radical adduct formation, or hydrogen atom transfer (Chen et al., 2014). For the selected bacillariophyte and haptophyte strains, under both exponential and early stationary phases, their high antioxidant capacity by carotenoids was significantly influenced by the presence of fucoxanthin and β-carotene. Fucoxanthin has higher antioxidant activity and sensitivity toward radicals than other carotenoids owing to the presence of the unique allenic carbon bond (C-7’) 5,6-monoepoxide, two hydroxyl groups, a carbonyl group, an acetyl group in the terminal ring, and six oxygen atoms in its molecular structure (Sachindra et al., 2007; Peng et al., 2011). Meanwhile, the antioxidant capacity of the chlorophyte strains was highly correlated with lutein and β-carotene, followed by neoxanthin and zeaxanthin content. In the stationary phase, their antioxidant capacity was largely influenced by violaxanthin, neoxanthin, and lutein, respectively. Similarly, the presence of the conjugated double bond in xanthophyll and carotenes such as β-carotene, vioxanthin, zeaxanthin, and other structurally related carotenoids enables them to be highly effective quenchers of singlet oxygen, which in algae, serves as part of their active defense mechanism against photooxidation (Stahl and Sies, 2003; Faraloni and Torzillo, 2017). While carotenoid compound accumulation is generally related to environmental conditions (i.e., light intensity), it has also been shown to be growth stage-dependent, mainly due to the effect of nutrient depletion on the later growth stages (Campo et al., 2007; McClure et al., 2018). Guo et al. (2016) reported an increase in fucoxanthin content at the beginning of cell growth and a decline in accumulation toward the end of the stationary phase due to nitrate depletion. Although the differences in carotenoid composition across division and growth phases have been previously reported, this study showed in detail what specific carotenoids are correlated with the antioxidant capacity under different growth phases.

Significant effects of phenolic compounds on the antioxidant activities of bacillariophytes and haptophytes were observed during the exponential phase, and for chlorophytes, during the exponential and stationary phases. Phenolic compounds have been linked to the antioxidative action in the biological system in several ways: as hydrogen-donating antioxidants reacting with RNS or ROS, breaking the cycle of new radical generation in the termination reaction, and also by chelating metal ions involved in free radical production (Pereira et al., 2009). It has been widely associated as one of the potential antioxidants in terrestrial plants for many years (Klejdus et al., 2010). However, its presence and contribution to the antioxidant capacity of microalgae have only recently been acknowledged; thus, data on this are still limited. Most previous studies focussed on chlorophytes, and there are still very few data available on bacillariophytes. Pereira et al. (2015) have reported the phenolic composition of several *Picochlorum* sp., *Desmochloris* sp., and *Nannochloris* sp. as being higher than that observed in several other species (5.8–114 mg GAE g^–1^ DW), except than *Picochlorum* sp., which has the highest phenolic content, specifically three phenolic acids (gallic, coumaric, and salicylic acids). Jerez-Martel et al. (2017) also observed high antioxidant activity in microalgal extracts rich in phenolic compounds, especially with high gallic and protocatechuic acid composition. Bulut et al. (2019) also found a significant correlation between antioxidant capacity and phenolic-rich *Scenedesmus* sp. ME02 extract (high in quercetin and rutin). In this study, we showed that phenolic compounds also contribute to the antioxidant activities of bacillariophytes and haptophytes. In addition, the contribution of phenolic content to the antioxidant capacity is particularly significant during the exponential phase. Because the phenolic compound accumulation is known to be growth-dependent, where their content is reduced under nutrient-limiting conditions (Goiris et al., 2012), the relative importance of phenolic content in antioxidant capacity in bacillariophytes and haptophytes may become higher during the exponential phase when carotenoid production decreases.

In the present study, the total fatty acid content, including PUFA, had little influence on the antioxidative capacity compared to carotenoid and phenolic content. This is contradictory to the reports that fatty acids and especially those with a high degree of unsaturation have been known to contribute to the antioxidant activity of various microalgae (de la Vega et al., 2011; Pereira et al., 2015; Banskota et al., 2018). However, when comparing each specific fatty acid compound, some significant relationships can be observed, especially during the exponential phase DPPH and O_2_^•–^, which are known to be effective in measuring the antioxidant capacity of PUFA, especially for the conjugated LA (Fagali and Catalá, 2008; Amaro et al., 2015). For bacillariophyte strains, high influence by LA, ALA, and DHA was observed toward the antioxidant capacity. Meanwhile, for chlorophyte strains, ARA, EPA, and LA had the highest influence on antioxidant capacity than other fatty acids. The differences in the influence of specific PUFAs on antioxidant capacity are highly dependent on the PUFA composition of each strain, which changes under different growth phases and has also been shown to be species-dependent (Mansour et al., 2005; Schwarzhans et al., 2015). To our knowledge, there are no other studies to investigate the PUFA composition and their relation to antioxidant capacity in detail under different growth phases. Our results clearly showed that the contribution of fatty acid to the antioxidant capacity in microalgae should be evaluated with each specific fatty acid compound but not a combined component like total PUFA or total fatty acids.

This study showed that the selected 10 tropical microalgal strains belonging to Bacillariophyta, Chlorophyta, and Haptophyta, have potential as a new source of natural antioxidants, along with a substantial amount of carotenoids, fatty acids, and phenolic compounds. In particular, carotenoid and phenolic content significantly influenced the antioxidant capacity of the selected strains. This study also suggests that the antioxidant capacity and related bioactive compound production of these strains are growth phase-dependent. The higher antioxidant capacity observed in the bacillariophytes and the haptophyte was majorly influenced by their phenolic content (in the exponential phase) and their dominant carotenoid, fucoxanthin, and β-carotene (under stationary phase), which may have been acting synergistically or antagonistically with other compounds. Meanwhile, the antioxidant capacity of chlorophytes during the exponential phase is largely influenced by the presence of the carotenoid lutein and β-carotene, and during the stationary phase, by violaxanthin, neoxanthin, and lutein. The high carotenoid, phenolic, and fatty acid contents still have the potential to be enhanced further for commercial purposes by cultivation under certain environmental or abiotic stressors. Moreover, our results revealed several high antioxidants producing candidate strains that can be exploited as feedstocks for various other applications, such as pharmaceutical, aquaculture, and biofuels, with their high nutritional value. These potential strains include *Thalassiosira weissflogii* (TRG10-P105) (high fucoxanthin), *Isochrysis galbana* (high fucoxanthin, fatty acid, and DHA content), *Amphora montana* (SLG4-03) (high fucoxanthin and ARA), *Chlorella sorokiniana* (SLG4-12) (high total PUFA), and *Tetraselmis suecica* (high phenolic, ALA).

## Data Availability Statement

The raw data supporting the conclusions of this article will be made available by the authors, without undue reservation.

## Author Contributions

NR, KT, and TK conceived and designed the experiments. NR collected and analyzed data, prepared the manuscript, and participated in the assembly and editing of the final manuscript. KT and TK supervised and assisted in the interpretation of data, assembly, and editing of the manuscript. TK, MW, NK, and HK assisted in collecting algal material and editing of the manuscript. YY assisted in the fatty acid analysis and the interpretation of the data. All authors contributed to the article and approved the submitted version.

## Conflict of Interest

The authors declare that the research was conducted in the absence of any commercial or financial relationships that could be construed as a potential conflict of interest.
