# Supplementary material for: Taxon- and Growth Phase-Specific Antioxidant Production by Chlorophyte, Bacillariophyte, and Haptophyte Strains Isolated From Tropical Waters
Source: Front Bioeng Biotechnol. 2020 Nov 23;8:581628. doi: 10.3389/fbioe.2020.581628 (PMC7719757; doi:10.3389/fbioe.2020.581628)
Supplement: Supplementary file 1 [file Table_1.DOCX]

Supplementary Material

## 1.0 Supplementary Table

**Supplementary Table 1.** The highest hits Blast score (against NCBI Nucleotide Collection (nr/nt) Database) of the sequenced isolate.

| **Division** | **Closest species (GeneBank)** | **Strain** |  | **Length (bp)** | **Coverage (%)** | **Identity (%)** | **Accession no** |
| --- | --- | --- | --- | --- | --- | --- | --- |
|  | *Nanochlorum eucaryotum* | SLG4-08 |  | 1743 | 99 | 99 | X06425 |
|  | *Nanochlorum eucaryotum* | SLG4-11 |  | 1552 | 100 | 100 | X06425 |
|  | *Picochlorum maculatum* | TRG9-05 |  | 2362 | 72 | 100 | KU561155 |
|  | *Picochlorum maculatum* | TRG9-06 |  | 1593 | 100 | 100 | KM055115 |
|  | *Chlorella sorokiniana* | SLG4-12 |  | 2288 | 83 | 99 | LK021940 |
|  | *Chlorella sorokiniana* | SLG4-13 |  | 1593 | 100 | 100 | MF101221 |
|  | *Oocystis heteromucosa* | TRG10-P102 |  | 1556 | 100 | 100 | KY013466 |
|  | *Oocystis marina* | TRG10-P104 |  | 1475 | 100 | 100 | MF100794 |
|  | *Chlamydomonas uva-maris* | SLG4-14 |  | 1081 | 99 | 98 | FR854390 |
| Bacillariophytes (Diatom) | *Amphora montana* | SLG4-03 |  | 1696 | 96 | 100 | KU561175 |
|  | *Amphora montana* | SLG4-17 |  | 1682 | 98 | 99 | KJ569265 |
|  | *Nitzschia capitellata* | TRG9-08 |  | 1034 | 100 | 100 | KT072978 |
|  | *Nitzschia capitellata* | TRG9-09 |  | 1053 | 99 | 100 | KT072978 |
|  | *Nitzschia palea* | SLG4-16 |  | 1654 | 98 | 95 | KY863477 |
|  | *Psammodictyon pustulatum* | TRG9-10 |  | 1050 | 99 | 99 | MH063502 |
|  | *Pauliella taeniata* | TRG8-02 |  | 1705 | 96 | 100 | KU561211 |
|  | *Navicula arenaria* | SLG4-18 |  | 1718 | 99 | 100 | KJ961668 |
|  | *Navicula radiosa* | TRG9-03 |  | 1743 | 98 | 99 | AM502027 |
|  | *Navicula arenaria* | SLG4-01 |  | 1734 | 99 | 100 | KJ961668 |
|  | *Navicula radiosa* | SLG4-02 |  | 1712 | 98 | 100 | AM501972 |
|  | *Thalassiosira weissflogii* | TRG10-P103 |  | 1453 | 99 | 99 | EF585582 |
|  | *Thalassiosira weissflogii* | TRG10-P105 |  | 1784 | 99 | 100 | EF585582 |

**Supplementary Table 2.** Carotenoid composition; results are express in mg g^−1^ dry weight (mean ± SE). Significantly high value among growth phases are printed in bold (*p <* 0.05), Exp: exponential phase, Sta: stationary phase.

| Strain | Growth phase | Fucoxanthin | Neoxanthin | Violaxanthin | Diadinoxanthin | Diatoxanthin | Zeaxanthin | Lutein | α carotene | β carotene | Total |
| --- | --- | --- | --- | --- | --- | --- | --- | --- | --- | --- | --- |
| *Tetraselmis suecica* | Exp | - | **0.98 ± 0.58** | **0.66 ± 0.16** | - | - | **0.06 ± 0.03** | **1.58 ± 0.17** | **0.22 ± 0.20** | **0.60 ± 0.10** | **3.94 ± 0.63** |
|  | Sta | - | 0.65 ± 0.14 | 0.33 ± 0.05 | - | - | 0.01 ± 0.00 | 0.51 ± 0.04 | 0.13 ± 0.04 | 0.23 ± 0.03 | 1.76 ± 0.24 |
| *Chlorella sorokiniana* SLG4-12 | Exp | - | 0.68 ± 0.50 | 0.57 ± 0.06 | - | - | 0.11 ± 0.01 | 3.73 ± 0.10 | - | 0.53 ± 0.08 | 5.70 ± 0.13 |
|  | Sta | - | 0.61 ± 0.49 | 0.45 ± 0.06 | - | - | 0.10 ± 0.01 | 3.78 ± 0.33 | - | 0.55 ± 0.08 | 5.59 ± 0.55 |
| *Nanochlorum eucaryotum* SLG4-08 | Exp | - | **1.23 ± 0.46** | **0.64 ± 0.24** | - | - | 0.07 ± 0.03 | **7.28 ± 2.78** | - | **1.41 ± 0.57** | **10.68 ± 4.09** |
|  | Sta | - | 0.55 ± 0.55 | 0.26 ± 0.10 | - | - | 0.08 ± 0.05 | 3.49 ± 0.90 | - | 0.60 ± 0.24 | 5.01 ± 1.39 |
| *Nanochlorum eucaryotum* SLG4-11 | Exp | - | **0.49 ± 0.16** | **0.26 ± 0.11** | - | - | 0.02 ± 0.01 | **0.92 ± 0.08** | **0.08 ± 0.04** | **0.24 ± 0.06** | 1.96 ± 0.26 |
|  | Sta | - | 0.11 ± 0.03 | 0.05 ± 0.03 | - | - | 0.02 ± 0.00 | 0.79 ± 0.02 | 0.01 ± 0.00 | 0.11 ± 0.04 | 1.09 ± 0.32 |
| *Chlorella sorokiniana* SLG4-13 | Exp | - | 0.25 ± 0.07 | 0.21 ± 0.06 | - | - | 0.04 ± 0.01 | **1.49 ± 0.47** | 0.01 ± 0.05 | **0.26 ± 0.09** | 2.27 ± 0.67 |
|  | Sta | - | 0.19 ± 0.03 | 0.21 ± 0.01 | - | - | 0.03 ± 0.00 | 0.82 ± 0.20 | 0.01 ± 0.01 | 0.18 ± 0.02 | 1.45 ± 0.26 |
| *Isochrysis galbana* | Exp | 10.65 ± 1.54 | - | - | **0.53 ± 0.14** | **0.91 ± 0.16** | - | - | - | 0.25 ± 0.02 | **15.66 ± 2.13** |
|  | Sta | **13.96 ± 1.91** | - | - | 0.37 ± 0.06 | 0.85 ± 0.25 | - | - | - | 0.20 ± 0.04 | 12.08 ± 1.80 |
| *Amphora montana* SLG4-03 | Exp | 10.44 ± 2.82 | - | - | 0.51 ± 0.14 | 0.47 ± 0.13 | - | - | - | 0.25 ± 0.08 | 11.66 ± 3.17 |
|  | Sta | **16.90 ± 2.75** | - | - | **0.91 ± 0.14** | **0.83 ± 0.22** | - | - | - | 0.33 ± 0.06 | **18.97 ± 3.12** |
| *Chaetoceros gracilis* | Exp | 7.83 ± 3.93 | - | - | 0.50 ± 0.28 | 0.26 ± 0.14 | - | - | - | 0.20 ± 0.10 | 8.80 ± 4.43 |
|  | Sta | **11.37 ± 2.11** | - | - | **0.69 ± 0.16** | **0.34 ± 0.15** | - | - | - | 0.26 ± 0.03 | **12.66 ± 2.45** |
| *Phaeodoctylum tricornutum* | Exp | 9.73 ± 0.41 | - | - | 0.80 ± 0.32 | 0.31 ± 0.02 | - | - | - | 0.23 ± 0.01 | 11.07 ± 0.45 |
|  | Sta | **12.34 ± 1.65** | - | - | **1.44 ± 0.18** | **0.40 ± 0.06** | - | - | - | 0.19 ± 0.05 | **14.37 ± 1.84** |
| *Thalassiosira weissflogii* TRG10-P103 | Exp | 3.24 ± 0.44 | - | - | **0.60 ± 0.17** | **0.23 ± 0.10** | - | - | - | **0.18 ± 0.05** | **7.14 ± 2.24** |
|  | Sta | **6.13 ± 1.92** | - | - | 0.17 ± 0.02 | 0.10 ± 0.02 | - | - | - | 0.09 ± 0.01 | 3.60 ± 0.49 |
| *Thalassiosira weissflogii* TRG10*-*P105 | Exp | 7.26 ± 0.87 | - | - | 0.73 ± 0.07 | 0.34 ± 0.06 | - | - | - | 0.29 ± 0.04 | 8.63 ± 1.04 |
|  | Sta | **10.29 ± 0.17** | - | - | **0.81 ± 0.02** | **0.61 ± 0.03** | - | - | - | **0.44 ± 0.03** | **12.15 ± 0.12** |
| Cymatosiraceae sp. TRG8-01 | Exp | 6.43 ± 1.13 | - | - | 0.22 ± 0.07 | 0.10 ± 0.03 | - | - | - | 0.01 ± 0.01 | 6.75 ± 1.22 |
|  | Sta | 7.45 ± 1.57 | - | - | **0.44 ± 0.07** | 0.06 ± 0.01 | - | - | - | 0.12 ± 0.04 | **8.07 ± 1.67** |

**Supplementary Table 3. ﻿**Mean fatty acid (FA) content (µg g^-1^ DW) of essential FAs, proportion of PUFAs, SFAs, MUFAs (in bracket % of total Fas) and FA totals (µg g^-1^ DW). Significantly high value among growth phases are printed in bold (*p <* 0.05), Exp: exponential phase, Sta: stationary phase.

| Strain | Growthphase | LA  (C18:2 n-6) | GLA  (C18:3 n-6) | ALA  (C18:3 n-3) | ARA  (C20:4 n-6) | EPA  (C20:5 n-3) | DHA (C22:6 n-3) | Total PUFA | Total SFA | Total  MUFA | Total  fatty acid |
| --- | --- | --- | --- | --- | --- | --- | --- | --- | --- | --- | --- |
| *Tetraselmis suecica* | Exp | **35.32 ± 5.80** | **1.35 ± 0.20** | **87.38 ± 15.17** | **3.19 ± 0.30** | **12.40 ± 1.44** | - | **251.2 (64.5)** | **69.45 (17.8)** | **69.25 (17.8)** | **389.45** |
|  | Sta | 19.29 ± 3.28 | 0.95 ± 0.16 | 46.11 ± 8.68 | 2.29 ± 0.24 | 8.70 ± 0.93 | - | 128.06 (51.8) | 59.38 (24.0) | 59.54 (24.1) | 246.99 |
| *Chlorella sorokiniana* SLG4-12 | Exp | **55.8 ± 5.99** | - | **71.72 ± 0.67** | - | 0.19 ± 0.20 | - | **343.45 (83.4)** | **53.03 (12.9)** | **15.49 (3.8)** | **411.96** |
|  | Sta | 20.70 ± 1.91 | - | 57.39 ± 5.51 | - | - | - | 139.2 (85.3) | 18.48 (11.3) | 5.54 (3.4) | 163.22 |
| *Nanochlorum eucaryotum* SLG4-08 | Exp | 77.21 ± 7.08 | - | **71.72 ± 6.69** | 0.29 ± 0.14 | 0.24 ± 0.24 | - | **233.89 (72.9)** | 69.04 (21.5) | 17.78 (5.5) | **320.72** |
|  | Sta | 76.62 ± 20.41 | - | 61.53 ± 16.86 | - | 0.30 ± 0.25 | - | 201.06 (64.1) | **89.01 (28.4)** | **23.42 (7.5)** | 313.49 |
| *Nanochlorum eucaryotum* SLG4-11 | Exp | **53.12 ± 0.74** | - | **91.02 ± 6.26** | 0.23 ± 0.11 | - | - | **221.24 (74.3)** | **65.45 (33.1)** | **11.24 (5.7)** | **197.92** |
|  | Sta | 31.14 ± 0.17 | - | 51.54 ± 6.87 | - | 0.20 ± 0.20 | - | 123.58 (70.0) | 48.47 (27.5) | 4.41 (2.5) | 176.46 |
| *Chlorella sorokiniana* SLG4-13 | Exp | 39.73 ± 2.06 | - | **61.11 ± 8.67** | - | - | - | 168.91 (64.5) | 41.7 (16.0) | **13.26 (5.1)** | **261.30** |
|  | Sta | **42.96 ± 8.41** | - | 55.11 ± 14.32 | - | - | - | **182.91 (81.7)** | **71.6 (32.0)** | 6.79 (3.0) | 223.77 |
| *Isochrysis galbana* | Exp | **29.82 ± 6.46** | **3.81 ± 1.27** | **64.75 ± 13.03** | 0.54 ± 0.11 | **5.15 ± 0.89** | **61.76 ± 9.37** | **364.33 (48.6)** | **194.93 (26.0)** | **189.99 (25.4)** | **749.25** |
|  | Sta | 18.87 ± 8.82 | 1.47 ± 0.69 | 33.93 ± 14.56 | **0.80 ± 0.41** | 4.71 ± 2.93 | 23.00 ± 4.30 | 194.64 (42.4) | 152.98 (33.3) | 111.46 (24.3) | 459.10 |
| *Amphora montana* SLG4-03 | Exp | 6.52 ± 2.38 | **2.34 ± 0.83** | **0.39 ± 0.06** | **25.08 ± 7.06** | **48.92 ± 39.89** | - | **122.48 (43.4)** | **84.03 (29.8)** | **75.68 (26.8)** | **282.19** |
|  | Sta | 5.05 ± 1.08 | 1.28 ± 0.88 | 0.22 ± 0.22 | 21.23 ± 1.85 | 38.71 ± 4.89 | - | 114.3 (46.7) | 70.28 (28.7) | 59.92 (24.5) | 244.51 |
| *Chaetoceros gracilis* | Exp | 0.96 ± 0.57 | 1.81 ± 1.04 | **3.64 ± 1.83** | 0.57 ± 0.57 | **94.93 ± 39.89** | - | **277.94 (64.1)** | 72.14 (16.6) | 83.55 (19.3) | **433.64** |
|  | Sta | 0.17 ± 0.17 | **2.23 ± 0.63** | 2.81 ± 0.61 | **1.18 ± 0.60** | 71.83 ± 11.13 | - | 179.18 (47.6) | **81.81 (22.6)** | **100.4 (27.8)** | 361.49 |
| *Phaeodoctylum tricornutum* | Exp | 7.25 ± 0.83 | - | 5.68 ± 0.48 | - | 82.29 ± 8.34 | - | **165.86 (59.8)** | 57.95 (20.9) | 53.51 (19.3) | 277.32 |
|  | Sta | 7.67 ± 0.29 | - | 5.21 ± 0.27 | - | 81.28 ± 5.30 | - | 155.71 (49.5) | **80.96 (25.7)** | **78.09 (24.8)** | **314.75** |
| *Thalassiosira weissflogii* TRG10-P103 | Exp | **5.72 ± 0.66** | 0.19 ± 0.19 | 1.97 ± 0.48 | - | **78.62 ± 1.06** | - | **281 (56.7)** | **98.45 (19.9)** | **116.01 (23.4)** | **495.46** |
|  | Sta | 1.24 ± 0.22 |  | 1.64 ± 0.11 | - | 19.68 ± 3.50 | - | 53.82 (10.9) | 18.81 (3.8) | 23.22 (4.7) | 100.85 |
| *Thalassiosira weissflogii* TRG10*-*P105 | Exp | **4.21 ± 1.20** | - | 2.19 ± 0.60 | - | **48.43 ± 6.21** | - | **176.05 (55.1)** | **74.38 (23.3)** | **69.00 (21.6)** | **319.40** |
|  | Sta | 3.05 ± 0.04 | - | **3.43 ± 0.10** | - | 37.75 ± 0.82 | - | 117.57 (36.8) | 57.67 (18.1) | 65.37 (20.5) | 240.61 |
| Cymatosiraceae sp. TRG8-01 | Exp | 6.29 ± 1.51 | 0.69 ± 0.17 | 0.68 ± 0.16 | 8.49 ± 1.63 | 42.59 ± 8.42 | - | 78.12 (24.3) | 73.69 (22.9) | **79.39 (24.7)** | **321.19** |
|  | Sta |  | - | - | 8.69 ± 0.38 | 44.42 ± 1.91 | - | 78.98 (32.2) | **88.71 (36.2)** | 77.52 (31.6) | 245.21 |

**2.0 Supplementary Figure**


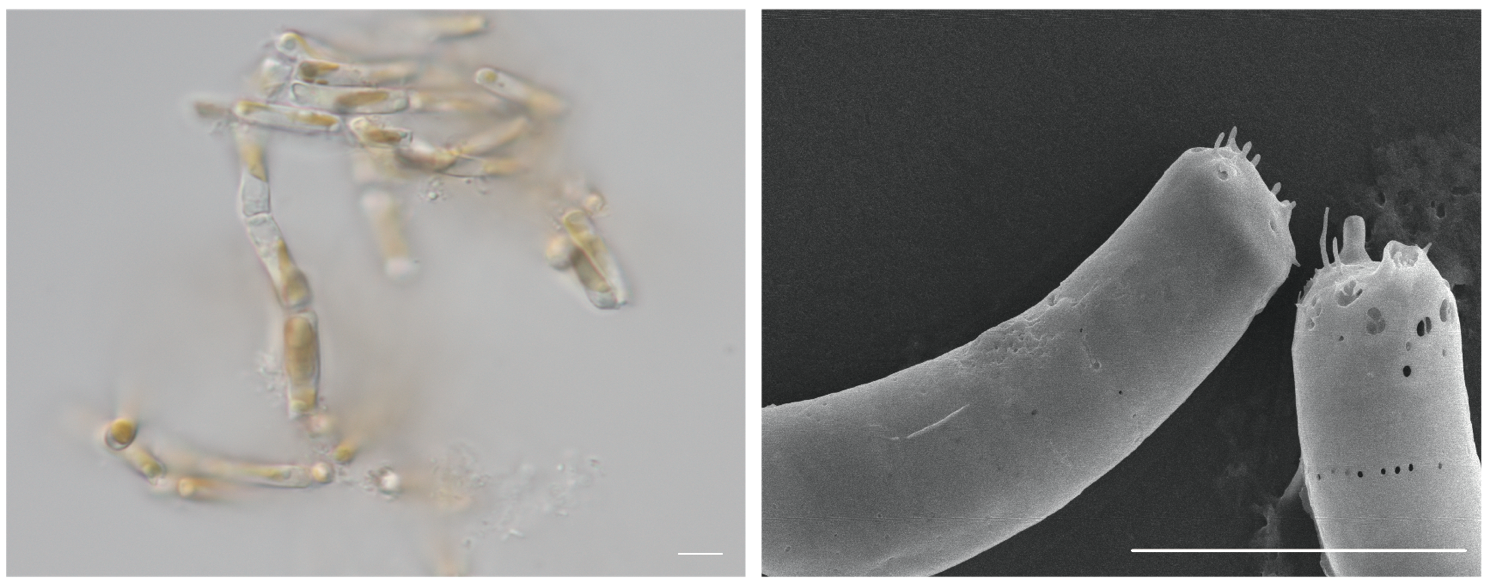


**Supplementary Figure 1. (A)** Light microscopy and (**B)** scanning electron microscopy (SEM) of diatom strain TRG8-01 showing cylindrical cells, frustules with areolae, processes, and occeluli. Scale bar = 3 µm for Figure 1A, 5 µm for Figure 1B. For light microscopy, live cells of TRG8-01 strain were observed under a Zeiss Axioskop 2 (Carl Zeiss, Göttingen, Germany) light microscope fitted with an epifluorescence device and equipped with an Axiocam HRc digital camera (Carl Zeiss). For SEM, cells of TRG8-01 strain were acid-cleaned with commercial sodium hypochlorite (6% NaOCl), centrifuged for 5 min at 5000 g, and the supernatant was removed. The cells were then rinsed with distilled water, centrifuged, and the supernatant was removed (repeated at least three times). The cells were put on an SEM plate coated with a poly-L-lysine and dried in a desiccator overnight before it was mounted onto an SEM stub, sputter-coated with platinum, and observed with an S-4800 (Hitachi, Tokyo, Japan) at an acceleration voltage of 5.0 kV.

Although the BLAST result showed that TRG8-01 as similar to bacillariophytes *Plagiogrammopsis vanheurckii* (99% similarity by ITS) and *Cymatosira belgica* (97% by LSU), it is obviously different morphologically (refer to Figure above). Therefore, the TRG8-01 is tentatively identified as Cymatosiraceae sp., referring to species within the Family Cymatosiraceae (bacillariophyte). This strains showed high total antioxidant capacity at 30.27 ± 1.16 (ABTS) and 16.80 ± 0.07 μmol trolox g^-1^ DW (DPPH), respectively. In addition, this strain also showed an substantial composition of carotenoid and fatty acid as described in Supplementary Table 2 and 3, with total phenolic content at 7.34 ± 1.34 (exponential phase) and 6.51 ± 0.71 mg GAE g^−1^ DW (stationary phase), respectively.
